# Gene Expression Changes Accompanying the Duodenal Adenoma-Carcinoma Sequence in Familial Adenomatous Polyposis

**DOI:** 10.14309/ctg.0000000000000053

**Published:** 2019-06-18

**Authors:** Sushrut S. Thiruvengadam, Margaret O'Malley, Lisa LaGuardia, Rocio Lopez, Zhen Wang, Bonnie L. Shadrach, Yanwen Chen, Chunbiao Li, Martina L. Veigl, Jill S. Barnholtz-Sloan, Rish K. Pai, James M. Church, Matthew F. Kalady, R. Matthew Walsh, Carol A. Burke

**Affiliations:** 1 Cleveland Clinic Lerner College of Medicine, Cleveland Clinic, Cleveland, Ohio, USA;; 2 Sanford R. Weiss MD Center for Hereditary Colorectal Neoplasia, Cleveland Clinic, Cleveland, Ohio, USA;; 3 Departments of Quantitative Health Science, Cleveland Clinic, Cleveland, Ohio, USA;; 4 Anatomic Pathology, Cleveland Clinic, Cleveland, Ohio, USA;; 5 Case Comprehensive Cancer Center, Case Western Reserve University, Cleveland, Ohio, USA;; 6 Colorectal Surgery, Cleveland Clinic, Cleveland, Ohio, USA;; 7 General Surgery, Cleveland Clinic, Cleveland, Ohio, USA;; 8 Gastroenterology and Hepatology, Cleveland Clinic, Cleveland, Ohio, USA.

## Abstract

Duodenal cancer in familial adenomatous polyposis (FAP) arises from adenomas. Differentially expressed genes (DEGs) in the duodenal adenoma-carcinoma pathway have been identified in murine FAP models, but similar data in patients with FAP are limited. Identifying such changes may have significance in understanding duodenal polyposis therapies and identifying cancer biomarkers. We performed a genome-wide transcriptional analysis to describe the duodenal adenoma-carcinoma sequence and determine changes distinguishing patients with FAP with and without duodenal cancer.

**METHODS:**

Transcriptional profiling was performed with the Affymetrix Human Transcriptome Array 2.0 on duodenal biopsies from 12 FAP patients with duodenal cancer (FAP cases) and 12 FAP patients without cancer (FAP controls). DEGs were compared between cancer-normal, adenoma-normal, and cancer-adenoma in FAP cases and between adenomas from FAP cases and FAP controls. Significant results at *P* < 0.05 were filtered using fold change > 2.

**RESULTS:**

Two hundred twenty-four DEGs were identified at an absolute fold change > 2. In adenoma-normal, downregulation of DEGs involved in metabolism of brush border proteins (*LCT*), lipids (*APOB/A4*), reactive oxygen species (*GSTA2*), and retinol (*RBP2*) was observed. In the cancer-adenoma comparison, upregulation of DEGs involved in cell invasion/migration (*POSTN, SPP1*) and downregulation of DEGs involved in Paneth differentiation (*DEFA5/6*) were observed. In the adenoma-adenoma comparison, downregulation of several DEGs (*CLCA1*, *ADH1C*, *ANXA10*) in FAP case adenomas was observed. DEGs with therapeutic potential include *SPP1*, which is involved in both cyclooxygenase and epidermal growth factor receptor pathways targeted by the sulindac/erlotinib combination for duodenal polyposis.

**DISCUSSION:**

We describe DEGs in the human duodenal adenoma-carcinoma sequence in FAP, which may have prognostic and therapeutic significance. Validation studies are needed to confirm these findings.

## INTRODUCTION

Familial adenomatous polyposis (FAP) is an autosomal dominant condition caused by loss-of-function in the adenomatous polyposis coli (*APC*) gene. The *APC* gene product inhibits Wnt/β-catenin signaling (1). In FAP, loss of function of *APC* results in promotion of β-catenin's tumorigenic effects and development of hundreds to thousands of intestinal adenomas. Resulting colorectal carcinoma (CRC) is nearly inevitable without early surgical intervention (2).

Duodenal cancer arises from duodenal adenomas and is a leading cause of death in FAP (3). Although the lifetime risk of duodenal polyposis in FAP approaches 100%, the cumulative incidence of cancer is 4.5% by the age of 57 (4). Chemoprevention with the cyclooxygenase 2 (COX-2) inhibitor celecoxib (5) and with a combination of the nonselective COX inhibitor sulindac and the epidermal growth factor receptor (EGFR) inhibitor erlotinib (6) have shown promise in decreasing polyp burden although long-term effect on cancer risk is unknown. Prophylactic duodenectomy is most effective at preventing cancer (7,8) but is associated with significant morbidity and mortality.

The Spigelman stage (SS) of duodenal polyposis (I-IV) is the only known tool to determine duodenal cancer risk and is used to guide endoscopic surveillance and need for prophylactic duodenectomy in FAP (4,9–11). Despite the prognostic value of SS, up to 40% of FAP patients with duodenal cancer do not have advanced SS polyposis and develop cancer while under surveillance (4,9,10). Therefore, it is clear that additional predictive factors must be identified.

Molecular characteristics of duodenal adenomas may aid in determining duodenal cancer risk. This is supported by gene expression studies on APC^Min/+^ mice, which, like patients with FAP, have a germline *APC* mutation but predominantly develop small intestinal polyposis (12). In these mice, normal intestine, adenoma, and carcinoma are distinguished by differentially expressed genes (DEGs) (13,14), suggesting that transcriptional changes herald malignant change of duodenal polyps in FAP. A recent study investigated gene expression changes between normal and adenomatous duodenal tissue in patients with FAP and found abnormalities in the Wnt/β-catenin, EGFR, and prostaglandin E2 (PGE2) pathways (15). However, no genome-wide investigation investigating the adenoma-carcinoma sequence in patients with FAP has been published. As a result, predictive and therapeutic targets to prevent duodenal cancer are largely unknown.

In this study, we first characterized the duodenal adenoma-carcinoma sequence in FAP by performing gene expression profiling on normal duodenum, adenoma, and cancer tissue from FAP patients with duodenal cancer (FAP cases). Next, we determined DEGs differentiating patients with duodenal cancer by comparing transcriptional profiles of adenomas from FAP cases with adenomas from FAP patients without cancer (FAP controls). Our ultimate objective was to uncover potential biomarkers for progression and therapeutic targets.

## METHODS

### Patient selection

Using the David G. Jagelman Inherited Colorectal Cancer Registries' Institutional Review Board-approved Cologene database and the Cleveland Clinic Anatomic Pathology database, we identified FAP patients with duodenal polyposis. Clinical and endoscopic characteristics were obtained from electronic and paper medical records. Pathology specimens were obtained from Anatomic Pathology archives.

**Figure 1.**
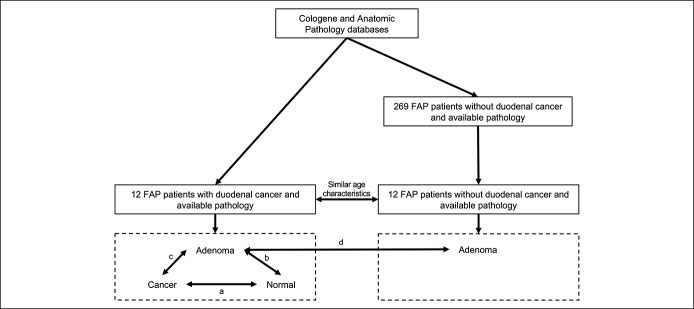
Patient selection and gene expression comparisons. Comparisons are labeled as follows: a) cancer-normal; b) adenoma-normal; c) cancer-adenoma; d) adenoma (from FAP cases)-adenoma (from FAP controls). FAP, familial adenomatous polyposis.

We identified 12 FAP patients with duodenal cancer (FAP cases) between 1988 and 2013 and 269 FAP patients with duodenal polyposis without cancer (FAP controls) undergoing upper endoscopic surveillance between 2005 and 2013. From this pool of FAP controls, we randomly selected 12 patients with similar age characteristics (mean, median, range) as our FAP cases (Figure 1). Clinical characteristics from FAP cases and FAP controls were collected, including age, gender, race, and sulindac or celecoxib use at the time of surveillance. Endoscopic characteristics were also collected, including polyp number (0–5, 6–20 or >20), size (0–5, 6–10 or >10 mm), histology (tubulous, tubulovillous, or villous) and dysplasia (low-grade or high-grade dysplasia) in the duodenum. Polyp histology and dysplasia information were taken from adenoma specimens obtained from FAP cases and FAP controls.

### Gene expression profiling

Upper endoscopic surveillance of patients with FAP was performed with a systematic approach (16). In each endoscopy, a forward-viewing and, when needed to view the papilla, side-viewing endoscope was used. Biopsies were performed on representative duodenal polyps and the papilla and specimens were preserved as formalin-fixed paraffin-embedded (FFPE) or Hollande's fixed samples. RNA extraction was performed with the Qiagen RNA FFPEasy kit. RNA was extracted from normal, adenoma, and cancer tissue from each of the 12 FAP cases and adenoma tissue from each of the 12 FAP controls, yielding 48 RNA samples. For adenoma samples, tissue with the most advanced histology was selected for extraction.

Gene expression profiling was performed using the Affymetrix GeneChip human transcriptome array (HTA) 2.0. Before profiling, quality control (QC) analysis verified that all 48 samples had sufficient yield (ranging from 43 to 159 μg). Our samples were run in 2 batches (24 samples per batch). RNA samples were converted to complementary DNA (cDNA) fragments, which were labeled to incorporate biotin. Labeled cDNA was then incubated with the HTA to allow hybridization of cDNA fragments to array oligonucleotides. Following hybridization, arrays underwent automated washing and fluorescent staining before collection of fluorescent signal intensities. At each step in this process, the same amount of RNA/cDNA from each sample was used to reduce batch-to-batch effects.

After raw data collection, each image file was visually inspected; no crude blemishes or grid misalignment was observed. Affymetrix proprietary algorithms featuring robust multi-chip analysis normalization was applied to all samples during data spreadsheet generation. A custom report monitoring 14 different QC metrics was generated using Affymetrix Expression Console. Principal among these was area under the curve (AUC), which indicated ease with which signal may be distinguished from background noise. AUC values range from 0 (imperfect) to 1 (perfect) and Affymetrix recommends values greater than 0.8. All 48 samples exceeded this metric; AUC values ranged from 0.899 to 0.979. Further corroboration of quality was indicated by consistency of other QC parameters, including perfect match mean and background mean. There were no outlier values, indicating no significant batch-to-batch discrepancy. Therefore, all 48 samples were retained in the data set. Transcription results have been deposited to NCBI GEO submission #GSE111156.

### Verification by quantitative PCR

qPCR was performed in triplicate using a TaqMan RNA-to-CT 1-Step Master Mixtures Kit with primers and monocolor hydrolysis probes, Hs009590101_m1 (*SPP1*), Hs00356112_m1 (*SI*), Hs0016636_m1 (*APOA4*), Hs00944023_M1 (*CEACAM5*), Hs01105012_m1 (*ANXA10*), and Hs00187842_m1 (*B2M*) (Applied Biosystems, Foster City, CA). qPCR was performed on an ABI PRISM 7500 real-time PCR system, according to the manufacturer's instructions. For all genes, qPCR cycling conditions were 48 °C for 15 minutes, 95 °C for 10 minutes, 50 cycles of 95 °C for 15 seconds, 60 °C for 1 minute, and 37 °C for 1 minute. PCR products were subjected to electrophoresis on an agarose gel to confirm absence of nonspecific PCR products. For each sample, the crossing threshold point (C_T_) for the amplification curves was determined by the second derivative maximum method. Absolute quantitation was performed with an in-run standard curve. The reference gene Beta-2 microglobulin (*B2M*) was used for separation of control populations and all results were normalized against calibrator RNA from MCF7. ∆CT was defined as C_T_ (candidate gene) − C_T_ (*B2M*). ∆∆CT was defined as ∆CT (candidate gene in sample RNA) − ∆CT (candidate gene in calibrator RNA). Relative expression value, or power, for each candidate gene in each sample was calculated as 2^−∆∆CT^.

### Statistical analysis

Analysis of transcriptional data was performed using the Affymetrix Expression Console Software package (version 1.3), R (version 3.2.0), and SAS (version 9.4). Raw data were processed using the Expression Console and further normalized with a cyclic loess approach. In comparisons, results for DEGs were expressed as either a positive fold change (FC), indicating upregulation or negative FC, indicating downregulation in the more advanced sample compared to the less advanced sample.

Within each FAP case, we performed comparisons between cancer and normal tissue (cancer-normal), between cancer and adenoma tissue (cancer-adenoma), and between adenoma and normal tissue (adenoma-normal) (Figure 1). For each pairwise comparison, we tested for significant differences at *P <* 0.05 using a nonparametric paired Wilcoxon test. To control for potential false positive results, we filtered pairwise results using a false discovery rate (FDR) < 0.10 and an absolute FC > 2 criteria. We then performed an unpaired comparison between adenomas from FAP cases and adenomas from FAP controls (adenoma-adenoma). We tested for significant differences at *P <* 0.05 using a nonparametric unpaired Wilcoxon test and filtered results using a FDR < 0.10 and an absolute FC > 2 criteria. Among DEGs in each comparison, we chose “representative” DEGs, which we defined as genes that have previously been implicated in FAP studies in mice or humans or in the development of other sporadic intestinal cancers.

Candidate DEGs were validated by quantitative polymerase chain reaction (PCR). For each comparisons, we tested for significant differences at *P <* 0.05 using nonparametric Wilcoxon tests.

## RESULTS

### Sample characteristics

The median age of FAP cases and FAP controls was 48.5 years (range 34–70 years). Clinical and endoscopic characteristics in FAP cases and FAP controls are described in Tables 1 and 2. Of 12 FAP cases, 4 had ampullary and 8 had nonampullary cancer. FAP cases and FAP controls did not differ with regard to age, gender, race, sulindac/celecoxib use nor did they differ with regard to polyp number, size, histology, or dysplasia (Table 2).

**Table 1.**
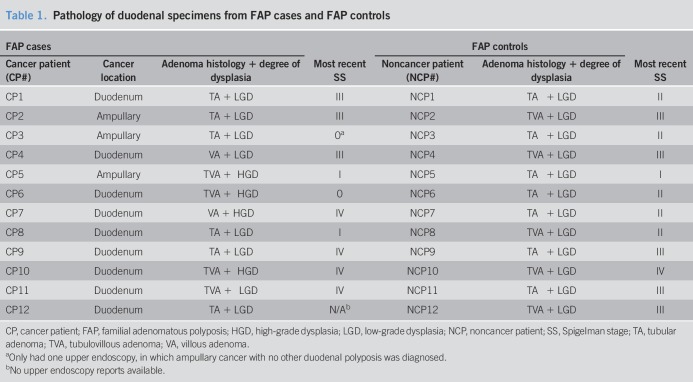
Pathology of duodenal specimens from FAP cases and FAP controls

**Table 2.**
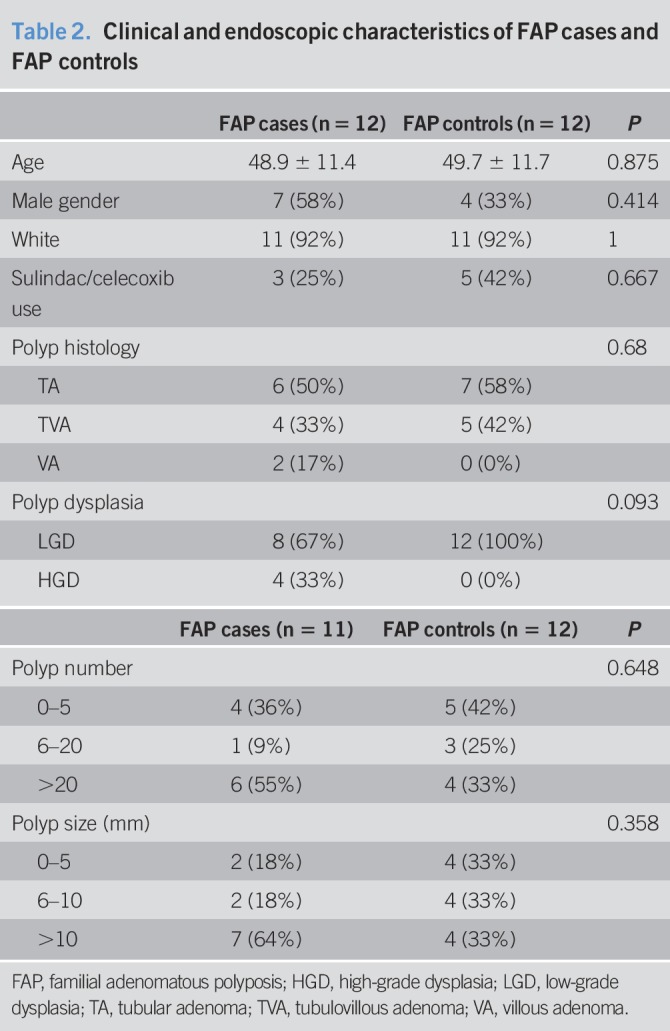
Clinical and endoscopic characteristics of FAP cases and FAP controls

### Overview of DEGs

**Figure 2.**
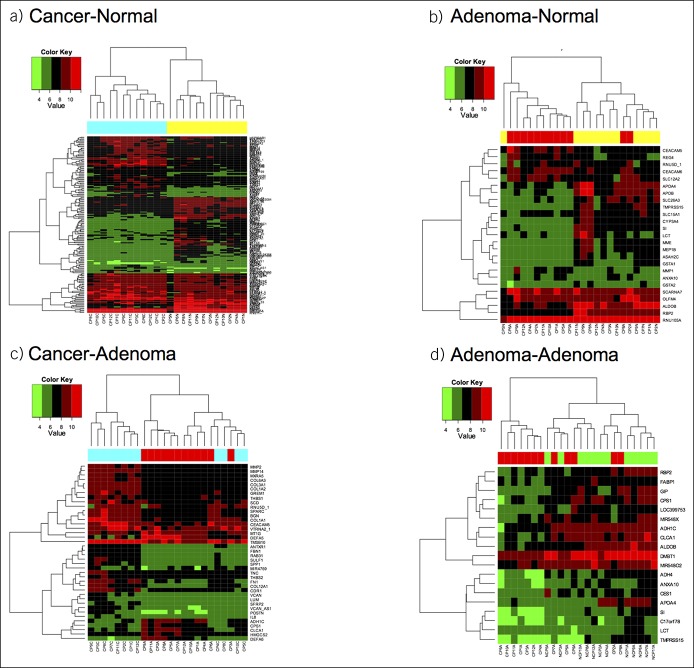
Hierarchical clustering for each of the 4 gene expression comparisons (**a**–**d**). DEGs with FC > 2 and *P* < 0.05 are shown, small nucleolar RNA, C/D box (SNORD) genes are not included. Each tissue type is color coded as follows: yellow = normal tissue from FAP case; red = adenoma from FAP case; cyan = cancer from FAP case; green = adenoma from FAP control. Lists of DEGs in each comparison is shown in Supplemental Table 2 (see Supplementary Digital content 1, http://links.lww.com/CTG/A51). DEG, differentially expressed gene; FAP, familial adenomatous polyposis; FC, fold change.

One hundred seventy-eight DEGs were identified with a FDR < 0.10 and an absolute FC > 2 in at least one comparison. Supplemental Table 1 (see Supplementary Digital Content 1, http://links.lww.com/CTG/A51) describes the number of DEGs in each comparison and Supplemental Table 2 (see Supplementary Digital Content 1, http://links.lww.com/CTG/A51) lists DEGs in each comparison. Protein-coding DEGs were classified into one of 9 groups according to cellular function/pathway of gene products. Table 3 shows representative DEGs within each group and FC in each comparison. Hierarchical clustering of DEGs in each comparison is shown in Figure 2.

**Table 3.**
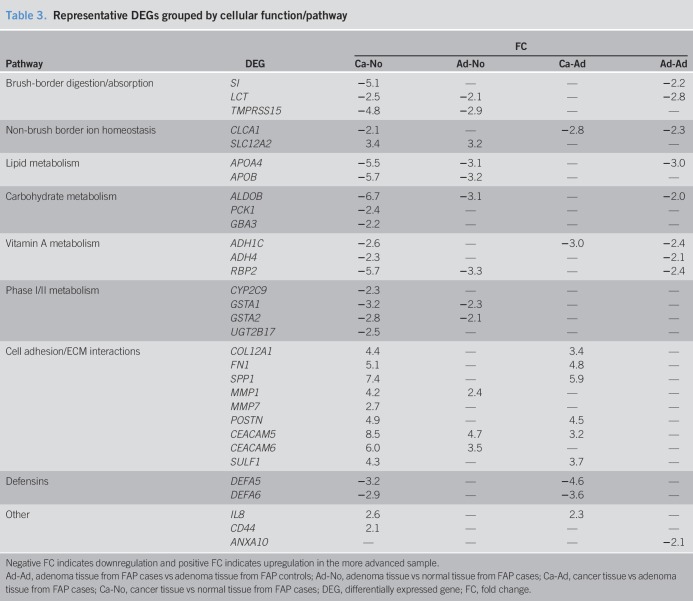
Representative DEGs grouped by cellular function/pathway

### Transition from normal duodenal to adenoma in FAP

In the adenoma-normal comparison, 19 protein-coding DEGs were identified. Neoplastic processes involving 8 representative DEGs are shown in Table 4.

**Table 4.**
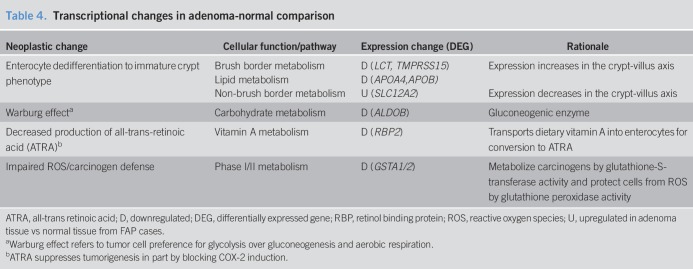
Transcriptional changes in adenoma-normal comparison

### Enterocyte dedifferentiation

Enterocyte dedifferentiation can be determined by examining expression along the crypt-villus axis and in the Caco-2 cell line, which spontaneously differentiates into mature small intestine (17). In adenoma-normal, we found downregulation of DEGs involved in brush-border metabolism. Among these, expression of *LCT* (18) and *TMPRSS15* (19) increases during the crypt-villus axis, while expression of *LCT* (20) increases with Caco-2 differentiation. We observed downregulation of *APOA4* and *APOB*, which encode apolipoproteins whose expression increases during Caco-2 cell differentiation (21). The downregulation of these brush border and lipid metabolism DEGs indicates enterocyte dedifferentiation. In adenoma-normal, we found upregulation of *SLC12A2,* which encodes the basolateral ion transporter NKCC1. NKCC1 expression *decreases* in the crypt-villus axis (22), suggesting that its upregulation further implicates enterocyte dedifferentiation.

### Warburg effect

During the transition from normal to adenoma, certain DEGs implicate the Warburg effect, in which proliferating tumor cells prefer glycolysis over gluconeogenesis and aerobic respiration. We found downregulation of *ALDOB*, which encodes a gluconeogenesis enzyme. Of note, *Aldob* is downregulated in adenomas of APC^Min/+^ mice (14).

### Decreased production of all-trans retinoic acid

In adenoma-normal, *RBP2* was downregulated. In small intestine, the retinol binding protein 2 (RBP2) mediates Vitamin A (retinol) uptake. Retinol is oxidized to all-*trans*-retinaldehyde by alcohol dehydrogenase and then to all-trans retinoic acid (ATRA) by aldehyde dehydrogenase (23). ATRA suppresses tumorigenesis in part by blocking induction of COX-2 (24). Therefore, downregulation of *RBP2* indicates that decreased ATRA production may play a role in the transition of normal duodenum to adenoma. Of note, decreased ATRA production is implicated in APC^Min/+^ mice adenomas, which show downregulation of *Adh1* (14).

### Impaired reactive oxygen species/carcinogen defense

In adenoma-normal, we found downregulation of *GSTA1* and *GSTA2*, which encode members of the α class of gluathione-S-transferase enzymes. These enzymes have glutathione peroxidase activity, which protects cells from reactive oxygen species (ROS). *GSTA1* downregulation is seen in normal duodenum from patients with FAP compared to non-FAP controls (25). Furthermore, downregulation of *Gsta4* is seen in intestinal adenomas of APC^Min/+^ mice (14). This suggests that diminished antioxidant defense plays a role in duodenal adenoma development in FAP.

### Transition from duodenal adenoma to cancer in FAP

In the cancer-adenoma comparison, there were 26 protein-coding DEGs. Neoplastic processes involving 8 representative DEGs are shown in Table 5.

**Table 5.**
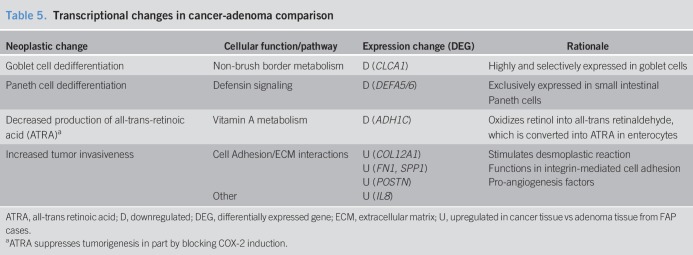
Transcriptional changes in cancer-adenoma comparison

### Goblet and Paneth cell dedifferentiation

In cancer-adenoma, we found downregulation of *CLCA1*, which encodes a chloride channel expressed in intestinal goblet cells. *CLCA1* expression increases with Caco-2 differentiation (26), suggesting that its downregulation may indicate goblet cell dedifferentiation.

In cancer-adenoma, we also found downregulation of *DEFA5* and *DEFA6*, which encode α-defensins and are primarily expressed in Paneth cells of the small intestine (27). Both are upregulated in colon adenomas and cancer compared to normal tissue (28), indicating abnormal Paneth cell differentiation in colon tumors (29). Given these findings, *DEFA5/6* downregulation may indicate Paneth dedifferentiation during the evolution of duodenal adenoma to cancer in FAP.

### Decreased production of ATRA

In cancer-adenoma, we found downregulation of *ADH1C*, which again implicates decreased ATRA production in the progression of duodenal neoplasia in FAP.

### Increased tumor invasiveness

In cancer-adenoma, upregulation of several DEGs involved in cell adhesion and extracellular matrix interactions was observed, including upregulation of *COL12A1*, which encodes for type XII collagen and is involved in the desmoplastic reaction between cancer cells and associated fibroblasts, which drives colon cancer metastases (30). Cancer tissue also exhibited upregulation of *FN1* and *SPP1*. *FN1* encodes fibronectin 1, which promotes cell proliferation and invasion by interacting with α5β1 integrin (31). *SPP1* encodes osteopontin (OPN), which mediates cell migration partially through interactions with αvβ3 integrin (32). In cancer-adenoma, *POSTN* and *IL8,* which encode the pro-angiogenesis factors periostin (33) and interleukin-8 (34), respectively, were also upregulated.

### DEGs with predictive potential in FAP

Among our representative DEGs, several have potential as tissue or serum biomarkers for progression of duodenal neoplasia.

### Potential tissue biomarkers for duodenal cancer in FAP

We identified 13 protein-coding DEGs that distinguished FAP case and FAP control adenomas, all of which were downregulated in FAP cases (Supplemental Table 1, see Supplementary Digital Content 1, http://links.lww.com/CTG/A51). Of these DEGs, *CLCA1*, *ADH1C*, and *ANXA10* have particular significance as potential tissue biomarkers.

*CLCA1* encodes a chloride channel expressed in goblet cells, whereas *ADH1C* encodes an alcohol dehydrogenase enzyme involved in retinol oxidation. Studies have shown *CLCA1* downregulation in CRC (26) and *ADH1C* downregulation in gastric cancer (23). In this study, *CLCA1* and *ADH1C* are downregulated in cancer compared to adenoma and in adenoma from FAP cases compared to FAP controls, indicating that downregulation of these DEGs within adenomas may indicate increased likelihood of neoplastic progression.

*ANXA10* encodes annexin 10, a calcium- and phospholipid-binding protein normally expressed in gastric mucosa that inhibits tumorigenesis by causing growth suppression and stimulation of apoptosis (35). Decreased ANXA10 expression is seen in gastric cancer (35). In this study, *ANXA10* expression followed a unique pattern. Within FAP cases, *ANXA10* does not differ in cancer-normal but is upregulated in adenoma-normal (FC 2.3, FDR 0.30) and downregulated in cancer-adenoma (FC −1.5, FDR < 0.10) comparisons. Furthermore, *ANXA10* is significantly downregulated in adenoma from FAP cases compared to FAP controls (FC −2.1, FDR < 0.10). Considering the aforementioned roles of *ANXA10*, it is possible that the upregulation of *ANXA10* in duodenal adenomas indicates a protective “gastric programming.” Downregulation during the transition from FAP control to FAP case adenoma and from FAP case adenoma to cancer may reflect a loss in the tumor suppressive function of *ANXA10*. Given these findings, determining tissue expression of *ANXA10* may predict the likelihood that a duodenal adenoma progresses to cancer in FAP.

### Potential serum biomarkers for duodenal cancer in FAP

Among the DEGs identified, *SPP1* and *CEACAM5* have potential as serum biomarkers for duodenal cancer in FAP.

*SPP1* encodes OPN. *SPP1* expression is 27-fold higher in sporadic ampullary cancer compared to normal duodenum and serum OPN progressively increases from healthy controls to patients with ampullary adenoma to patients with sporadic ampullary cancer (36). *CEACAM5* encodes membrane-bound and secreted carcinoembryonic antigen (CEA). For CRC, serum CEA is an independent prognostic factor for recurrence and survival after curative resection (37). In this study, *CEACAM5* is the only DEG upregulated in the adenoma-normal and cancer-adenoma comparisons (Table 3). Together, these findings suggest that serum OPN and CEA may help determine development of duodenal polyposis and progression to duodenal cancer in FAP.

### DEGs with therapeutic potential in FAP

Certain DEGs may have significance in existing and novel chemopreventive therapies for duodenal polyposis.

Both celecoxib (5) and the sulindac/erlotinib combination (38) decrease duodenal polyp burden in FAP. We found upregulation of *SPP1* in cancer-normal and cancer-adenoma comparisons. *SPP1* is a Wnt/β-catenin target gene (39) and administration of the COX-2 inhibitor parecoxib to APC^∆14/+^ mice, which display a FAP phenotype, downregulates *SPP1* by inhibition of Wnt/β-catenin while decreasing intestinal tumor load and mice morality (40). OPN is an upstream activator of the EGFR pathway (41). In non-small-cell lung cancer cell lines, the radiosensitizing effect of erlotinib is abolished after OPN depletion (42). Given its role as a target of PGE2 signaling and an activator of EGFR signaling, tissue levels of OPN may be of particular significance in predicting response to the sulindac/erlotinib combination regimen.

*CEACAM6* upregulation in the adenoma-normal and cancer-normal comparisons was also noted. *CEACAM6* encodes a membrane-bound cell adhesion molecule, which confers resistance to anoikis, the apoptosis induced by lack of correct cell/extracellular matrix attachment (43). This allows for cancer cell survival and invasiveness. Accordingly, *CEACAM6* overexpression is seen in CRC (44) and pancreatic cancer (45). In a murine model of pancreatic cancer, administration of a CEACAM6-specific monoclonal antibody conjugated with immunotoxin increases tumor apoptosis and decreases tumor growth (46). In nonhuman primates, this antibody-drug conjugate has minimal toxicity, with a dose-dependent, reversible neutropenia (47). These findings implicate *CEACAM6* as a potential novel therapeutic target in the treatment of duodenal polyposis in FAP.

### PCR verification

We determined expression levels of candidate genes *SPP1*, *CEACAM5*, *SI*, *APOA4*, and *ANXA10* by PCR. In certain samples from certain patients, PCR could not be successfully performed and expression levels were undefined (Supplemental Table 3, see Supplementary Digital Content 1, http://links.lww.com/CTG/A51).

For cancer-normal and cancer-adenoma comparisons, the sample size for comparison of *SI* expression was very low (n = 5). Therefore, we decided to exclude PCR results from *SI* expression. For the remaining genes, FAP cases 10 and 11 consistently did not yield results on PCR. Both FAP cases 10 and 11 had samples preserved with Hollande's fixative (Supplemental Table 4, see Supplementary Digital Content 1, http://links.lww.com/CTG/A51), which can affect RNA yield and quality (48) and therefore may explain the failure of PCR expression analysis in these samples.

Table 6 shows HTA and PCR results. For every comparison, direction of FC mirrored HTA findings. Specific magnitude of FC and statistical significance is detailed below.

1. *SPP1:* Gene expression differences in *SPP1* was fully verified by PCR.
2. *CEACAM5:* PCR analyses verified no difference in adenoma-adenoma comparison. As in HTA analysis, PCR analysis showed upregulation in cancer-normal and cancer-adenoma, but each comparison had a trend toward significance.
3. *APOA4:* PCR analysis verified downregulation in all comparisons; of note, for adenoma-normal, PCR analysis showed a trend toward downregulation.
4. *ANXA10*: PCR analysis verified *ANXA10* upregulation in adenoma-normal, downregulation in cancer-adenoma, and the lack of significant difference in cancer-normal. In adenoma-adenoma, PCR analysis showed a trend toward downregulation, which mirrored significant HTA results.

**Table 6.**
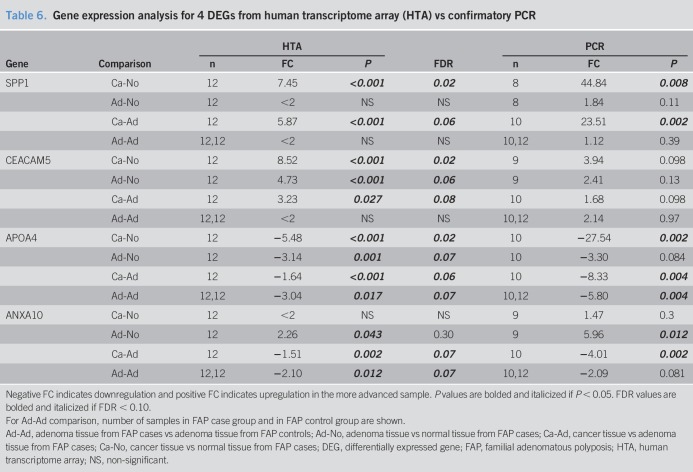
Gene expression analysis for 4 DEGs from human transcriptome array (HTA) vs confirmatory PCR

## DISCUSSION

Duodenal cancer is a leading cause of death in FAP after colectomy. SS IV duodenal polyposis is a risk factor for duodenal cancer, yet many FAP patients have no history of SS IV polyposis (4,9,10), indicating a need for additional predictors of cancer risk. In APC^Min/+^ mice, gene expression changes accompany the evolution of small intestinal neoplasia (13,14). To date, no such genome-wide investigation has been performed in patients with FAP. In this study, we described the duodenal adenoma-carcinoma sequence in FAP by comparing normal, adenoma, and cancer tissue of 12 duodenal cancer cases. In the transition from normal duodenum to adenoma, we found potential roles for enterocyte dedifferentiation, the Warburg effect, decreased ATRA synthesis, and impaired ROS/carcinogen defense. In the transition from adenoma to cancer, Paneth/goblet cell dedifferentiation, decreased ATRA synthesis, and increased tumor invasiveness were implicated.

Several DEGs distinguished FAP case from FAP control adenomas. *ANXA10* is unique in that it is upregulated from normal to adenoma in FAP cases but downregulated from FAP case adenoma to cancer and from FAP control adenoma to FAP case adenoma. Given its function, *ANXA10* expression in adenomas may indicate a protective “gastric programming” that suppresses neoplastic evolution. We also identified DEGs upregulated in cancer compared to adenoma that may have utility as biomarkers for neoplastic progression, including *SPP1* and *CEACAM5* (36,49,50).

Delker et al. (15) performed gene expression analysis on normal duodenum and adenoma in patients with FAP who were either treated with sulindac/erlotinib or with placebo. In the placebo group, they performed an adenoma-normal comparison similar to the one performed in this study. Genes involved in Wnt, PGE2, and EGFR signaling were differentially expressed in the placebo group but not in the sulindac/erlotinib group, indicating a beneficial inhibition of these pathways (15). Duodenal polyps in this study also exhibited upregulation of *CD44*, a cancer stem cell marker associated with PGE2 signaling (51), and *MMP7*, which encodes a matrix metalloproteinase and is a Wnt/Beta-catenin signaling target (52). In our study, *CD44* and *MMP7* were both upregulated in our cancer-normal comparisons (Table 3). Furthermore, *MMP1*, which is also a WNT/Beta-catenin target (53), was upregulated in our adenoma-normal and cancer-normal comparisons (Table 3).

We also identified DEGs with therapeutic potential in FAP. We found upregulation of *SPP1*, which plays a role in both the tumorigenic effect of PGE2 (40) and in activation of EGFR signaling (41). Given its relation to both pathways, determining *SPP1* expression may help predict response to sulindac/erlotinib therapy. We also identified *CEACAM6* as a potential novel therapeutic target for duodenal polyposis control in FAP. CEACAM6 has been successfully targeted in animal models of pancreatic cancer (45,47).

Several limitations merit further discussion. Our RNA extraction and gene expression profiling procedures were specific for FFPE and Hollande's fixatives and all RNA samples met QC checkpoints for HTA profiling. However, during PCR verification, several samples, particularly Hollande's fixed samples, yielded undefined results. As a result, PCR comparisons involved lower sample sizes and, while FCs matched our HTA results for 4 candidate genes, *P* values in some comparisons did not reach statistical significance. This indicates the importance of future validation studies with independent cohorts. Another limitation is the potential for false positives. To address this, we applied a FDR < 0.10 cutoff for our DEGs. Although there is still potential for false positives despite this cutoff, it should be noted that of 52 DEGs that differed in 2+ comparisons, all 52 differed in the same direction (upregulation/downregulation) in each comparison. Similarly, of 8 DEGs that differed in 3+ comparisons, all differed in the same direction in each comparison.

In summary, we have conducted the first ever genome-wide expression analysis of duodenal neoplasia in FAP. Future validation studies with immunohistochemical staining or Western Blot analysis are needed to verify protein expression of candidate genes. Furthermore, for genes whose expression may predict response to celecoxib or sulindac/erlotinib therapy, gene knock-in or knock-out in APC^Min/+^ mice can be performed to determine effect on therapeutic response. Effect of the CEACAM6 antibody-drug conjugate on APC^Min/+^ mice can also be investigated, and if this shows therapeutic benefit and low toxicity, targeting CEACAM6 may emerge as a viable option for duodenal polyposis control in FAP.

## CONFLICTS OF INTEREST

**Guarantor of the article:** Sushrut S. Thiruvengadam, MD.

**Specific author contributions:** Study concept and design: S.S.T. and C.A.B. Acquisition of data (retrospective identification and selection of patients): S.S.T., R.L., M.O. and L.L. Acquisition of data (identification and preparation of archived tissue samples for transcriptional profiling): S.S.T. and R.K.P. Acquisition of data (transcriptional profiling): M.L.V. and C.L. Acquisition of data (PCR verification): S.S.T., Z.W., and B.S. Analysis and interpretation of the data: S.S.T., R.L., Y.C., J.S.B., and C.A.B. Drafting of the manuscript: S.S.T. Critical revision of the manuscript for important intellectual content: C.A.B., J.B.S., M.L.V., B.S., and Z.W. All authors have reviewed the final submitted draft.

**Financial support:** Grant support provided by Cleveland Clinic Research Program Committees Award (RPC 2014–1047). Technical support for this work was provided by the Gene Expression and Genotyping Facility, a component of the Integrated Genomic Shared Resource sponsored by the Case Comprehensive Cancer Center (P30 CA43703). This work was independent of this funding.

**Potential competing interests:** C.A.B. reports the following relevant financial disclosures: grants from Cancer Prevention Pharmaceuticals, Ferring Pharmaceuticals, consultant royalties and personal fees from Sucampo, Aries, and Salix Pharmaceuticals. M.K. reports the following relevant financial disclosures: consulting honorarium from Helomics and TransEnterix. The other authors affirm that they have no relevant financial or personal conflicts to disclose.

**Study Highlights**

**WHAT IS KNOWN**

✓ Murine models of FAP have identified DEGs in the duodenal adenoma-carcinoma sequence.
✓ This has not been studied in patients with FAP.

**WHAT IS NEW HERE**

✓ Transition from normal duodenum to adenoma is characterized by abnormal metabolism of brush border proteins, lipids, ROS, and retinol and transition from adenoma to cancer was characterized by upregulation of DEGs involved in cell invasion and migration.
✓ Certain DEGs differed between adenomas from cancer patients and controls.
✓ Several DEGs have potential therapeutic significance in existing chemopreventive regimens, including the sulindac/erlotinib combination for duodenal polyposis in FAP.

**TRANSLATIONAL IMPACT**

✓ In the future, physicians may be able to use differential expression of certain genes in order to determine progression of duodenal adenoma to cancer in FAP.
✓ In the future, physicians may be able to target novel and existing chemopreventive pathways to prevent progression of duodenal polyposis and development of cancer in FAP.

## Supplementary Material

SUPPLEMENTARY MATERIAL
