# Supplementary material for: Gene Expression Changes Accompanying the Duodenal Adenoma-Carcinoma Sequence in Familial Adenomatous Polyposis
Source: Clin Transl Gastroenterol. 2019 Jun 18;10(6):e00053. doi: 10.14309/ctg.0000000000000053 (PMC6613862; doi:10.14309/ctg.0000000000000053)
Supplement: SUPPLEMENTARY MATERIAL [file ct9-10-e00053-s001.docx]

| **Table 1: Pathology of Duodenal Specimens from FAP Cases and FAP Controls** | | | | | | |
| --- | --- | --- | --- | --- | --- | --- |
| **FAP Cases** | | |  | **FAP Controls** | |  |
| **Cancer Patient (CP#)** | **Cancer Location** | **Adenoma Histology + Degree of Dysplasia** | **Most Recent Spigelman Stage** | **Non-Cancer Patient (NCP#)** | **Adenoma Histology + Degree of Dysplasia** | **Spigelman Stage** |
| **CP1** | Duodenum | TA + LGD | III | **NCP1** | TA + LGD | II |
| **CP2** | Ampullary | TA + LGD | III | **NCP2** | TVA + LGD | III |
| **CP3** | Ampullary | TA + LGD | 0^a^ | **NCP3** | TA + LGD | II |
| **CP4** | Duodenum | VA + LGD | III | **NCP4** | TVA + LGD | III |
| **CP5** | Ampullary | TVA + HGD | I | **NCP5** | TA + LGD | I |
| **CP6** | Duodenum | TVA + HGD | 0 | **NCP6** | TA + LGD | II |
| **CP7** | Duodenum | VA + HGD | IV | **NCP7** | TA + LGD | II |
| **CP8** | Duodenum | TA + LGD | I | **NCP8** | TVA + LGD | II |
| **CP9** | Duodenum | TA + LGD | IV | **NCP9** | TA + LGD | III |
| **CP10** | Duodenum | TVA + HGD | IV | **NCP10** | TVA + LGD | IV |
| **CP11** | Duodenum | TVA + LGD | IV | **NCP11** | TA + LGD | III |
| **CP12** | Duodenum | TA + LGD | N/A^b^ | **NCP12** | TVA + LGD | III |
| TA= Tubular, TVA = Tubulovillous, VA= Villous Adenoma, LGD= low-grade, HGD= high-grade dysplasia. ^a^Only had one EGD, in which ampullary cancer with no other duodenal polyposis was diagnosed. ^b^No EGD reports available | | | | | | |

| **Table 2: Clinical and Endoscopic Characteristics of FAP Cases and FAP Controls** | | | | |
| --- | --- | --- | --- | --- |
|  |  |  |  |  |
|  | | **FAP Cases (n=12)** | **FAP Controls (n=12)** | ***P*** |
| **Age** | | 48.9 +/- 11.4 | 49.7 +/- 11.7 | 0.875 |
| **Male Gender** | | 7(58%) | 4(33%) | 0.414 |
| **White** | | 11(92%) | 11(92%) | 1 |
| **Sulindac/Celecoxib use** | | 3(25%) | 5(42%) | 0.667 |
| **Polyp Histology** | **TA** | 6 (50%) | 7 (58%) | 0.68 |
|  | **TVA** | 4 (33%) | 5 (42%) |  |
|  | **VA** | 2 (17%) | 0 (0%) |  |
| **Polyp Dysplasia** | **LGD** | 8 (67%) | 12 (100%) | 0.093 |
|  | **HGD** | 4 (33%) | 0 (0%) |  |
|  |  | **FAP Cases (n=11)** | **FAP Controls (n=12)** | ***P*** |
| **Polyp Number** | **0 to 5** | 4(36%) | 5(42%) | 0.648 |
|  | **6 to 20** | 1(9%) | 3(25%) |  |
|  | **>20** | 6(55%) | 4(33%) |  |
| **Polyp Size (mm)** | **0 to 5** | 2(18%) | 4(33%) | 0.358 |
|  | **6 to 10** | 2(18%) | 4(33%) |  |
|  | **>10** | 7(64%) | 4(33%) |  |
| TA= Tubular, TVA = Tubulovillous, VA= Villous Adenoma, LGD= low-grade, HGD= high-grade dysplasia. One FAP case did not have endoscopy report with polyp number or size available, so analysis was performed on the remaining 11 FAP cases | | | | |

| **Table 3: Representative DEGs Grouped by Cellular Function/Pathway** | | | | | |
| --- | --- | --- | --- | --- | --- |
|  |  |  |  |  |  |
| **Pathway** | **DEG** | **Fold Change** | | | |
|  |  | **Ca-No** | **Ad-No** | **Ca-Ad** | **Ad-Ad** |
| **Brush-Border Digestion/Absorption** | *SI* | -5.1 | - | - | -2.2 |
|  | *LCT* | -2.5 | -2.1 | - | -2.8 |
|  | *TMPRSS15* | -4.8 | -2.9 | - | - |
| **Non-Brush Border Ion Homeostasis** | *CLCA1* | -2.1 | - | -2.8 | -2.3 |
|  | *SLC12A2* | 3.4 | 3.2 | - | - |
| **Lipid Metabolism** | *APOA4* | -5.5 | -3.1 | - | -3.0 |
|  | *APOB* | -5.7 | -3.2 | - | - |
| **Carbohydrate Metabolism** | *ALDOB* | -6.7 | -3.1 | - | -2.0 |
|  | *PCK1* | -2.4 | - | - | - |
|  | *GBA3* | -2.2 | - | - | - |
| **Vitamin A Metabolism** | *ADH1C* | -2.6 | - | -3.0 | -2.4 |
|  | *ADH4* | -2.3 | - | - | -2.1 |
|  | *RBP2* | -5.7 | -3.3 | - | -2.4 |
| **Phase I/II Metabolism** | *CYP2C9* | -2.3 | - | - | - |
|  | *GSTA1* | -3.2 | -2.3 | - | - |
|  | *GSTA2* | -2.8 | -2.1 | - | - |
|  | *UGT2B17* | -2.5 | - | - | - |
| **Cell Adhesion/ECM Interactions** | *COL12A1* | 4.4 | - | 3.4 | - |
|  | *FN1* | 5.1 | - | 4.8 | - |
|  | *SPP1* | 7.4 | - | 5.9 | - |
|  | *MMP1* | 4.2 | 2.4 | - | - |
|  | *MMP7* | 2.7 | - | - | - |
|  | *POSTN* | 4.9 | - | 4.5 | - |
|  | *CEACAM5* | 8.5 | 4.7 | 3.2 | - |
|  | *CEACAM6* | 6.0 | 3.5 | - | - |
|  | *SULF1* | 4.3 | - | 3.7 | - |
| **Defensins** | *DEFA5* | -3.2 | - | -4.6 | - |
|  | *DEFA6* | -2.9 | - | -3.6 | - |
| **Other** | *IL8* | 2.6 | - | 2.3 | - |
|  | *CD44* | 2.1 | - | - | - |
|  | *ANXA10* | - | - | - | -2.1 |
| Negative fold change indicates downregulation and positive fold change indicates upregulation in the more advanced sample. Ca-No= Cancer tissue vs Normal tissue from FAP cases; Ad-No= Adenoma tissue vs Normal tissue from FAP cases; Ca-Ad= Cancer tissue vs Adenoma tissue from FAP cases; Ad-Ad= Adenoma tissue from FAP cases vs Adenoma tissue from FAP controls | | | | | |

| **Table 4: Transcriptional Changes in Adenoma-Normal Comparison** | | | |
| --- | --- | --- | --- |
|  |  |  |  |
| **Neoplastic Change** | **Cellular Function/Pathway** | **Expression Change (DEG)** | **Rationale** |
| **Enterocyte de-differentiation to immature crypt phenotype** | Brush Border Metabolism | D (*LCT, TMPRSS15*) | Expression increases in the crypt-villus axis |
|  | Lipid Metabolism | D (*APOA4,APOB)* |  |
|  | Non-BB Metabolism | U (*SLC12A2*) | Expression decreases in the crypt-villus axis |
| **Warburg Effect^a^** | Carbohydrate Metabolism | D (*ALDOB*) | Gluconeogenic enzyme |
| **Decreased production of all-trans-retinoic acid (ATRA)^b^** | Vitamin A Metabolism | D (*RBP2*) | Transports dietary vitamin A into enterocytes for conversion to ATRA. |
| **Impaired ROS/carcinogen defense** | Phase I/II Metabolism | D (*GSTA1/2*) | Metabolize carcinogens via glutathione-S-transferase activity and protect cells from ROS via glutathione peroxidase activity |
| D=Downregulated; U=Upregulated in adenoma tissue vs normal tissue from FAP cases. ^a^Warburg Effect refers to tumor cell preference for glycolysis over gluconeogenesis and aerobic respiration. ^b^ATRA suppresses tumorigenesis in part by blocking COX-2 induction | | | |

| **Table 5: Transcriptional Changes in Cancer-Adenoma Comparison** | | | |
| --- | --- | --- | --- |
|  |  |  |  |
| **Neoplastic Change** | **Cellular Function/Pathway** | **Expression Change (DEG)** | **Rationale** |
| **Goblet cell de-differentiation** | Non-BB Metabolism | D (*CLCA1*) | Highly and selectively expressed in goblet cells |
| **Paneth cell de-differentiation** | Defensin Signaling | D (*DEFA5/6*) | Exclusively expressed in small intestinal Paneth cells |
| **Decreased production of all-trans-retinoic acid (ATRA)^a^** | Vitamin A Metabolism | D (*ADH1C*) | Oxidizes retinol into all-trans retinaldehyde, which is converted into ATRA in enterocytes. |
| **Increased tumor invasiveness** | Cell Adhesion/ECM Interactions | U (*COL12A1*) | Stimulates desmoplastic reaction |
|  |  | U (*FN1, SPP1*) | Functions in integrin-mediated cell adhesion |
|  |  | U (*POSTN*) | Pro-angiogenesis factors |
|  | Other | U(*IL8*) |  |
| D=Downregulated; U=Upregulated in cancer tissue vs adenoma tissue from FAP cases. ^a^ATRA suppresses tumorigenesis in part by blocking COX-2 induction. | | | |

| **Table 6: Gene expression analysis for 4 DEGs from Human Transcriptome Array (HTA) vs confirmatory PCR** | | | | | | | | |
| --- | --- | --- | --- | --- | --- | --- | --- | --- |
|  |  |  |  |  |  |  |  |  |
|  |  | **HTA array** | | |  | **PCR** | | |
| **Gene** | **Comparison** | **n** | **AFC** | ***P*** | **FDR** | **n** | **AFC** | ***P*** |
| *SPP1* | Ca-No | 12 | 7.45 | ***<.001*** | ***0.02*** | 8 | 44.84 | ***0.008*** |
|  | Ad-No | 12 | <2 | NS | NS | 8 | 1.84 | 0.11 |
|  | Ca-Ad | 12 | 5.87 | ***<.001*** | ***0.06*** | 10 | 23.51 | ***0.002*** |
|  | Ad-Ad | 12,12 | <2 | NS | NS | 10,12 | 1.12 | 0.39 |
| *CEACAM5* | Ca-No | 12 | 8.52 | ***<.001*** | ***0.02*** | 9 | 3.94 | 0.098 |
|  | Ad-No | 12 | 4.73 | ***<.001*** | ***0.06*** | 9 | 2.41 | 0.13 |
|  | Ca-Ad | 12 | 3.23 | ***0.027*** | ***0.08*** | 10 | 1.68 | 0.098 |
|  | Ad-Ad | 12,12 | <2 | NS | NS | 10,12 | 2.14 | 0.97 |
| *APOA4* | Ca-No | 12 | -5.48 | ***<0.001*** | ***0.02*** | 10 | -27.54 | ***0.002*** |
|  | Ad-No | 12 | -3.14 | ***0.001*** | ***0.07*** | 10 | -3.30 | 0.084 |
|  | Ca-Ad | 12 | -1.64 | ***<0.001*** | ***0.06*** | 10 | -8.33 | ***0.004*** |
|  | Ad-Ad | 12,12 | -3.04 | ***0.017*** | ***0.07*** | 10,12 | -5.80 | ***0.004*** |
| *ANXA10* | Ca-No | 12 | <2 | NS | NS | 9 | 1.47 | 0.3 |
|  | Ad-No | 12 | 2.26 | ***0.043*** | 0.30 | 9 | 5.96 | ***0.012*** |
|  | Ca-Ad | 12 | -1.51 | ***0.002*** | ***0.07*** | 10 | -4.01 | ***0.002*** |
|  | Ad-Ad | 12,12 | -2.10 | ***0.012*** | ***0.07*** | 10,12 | -2.09 | 0.081 |
| Negative fold change indicates downregulation and positive fold change indicates upregulation in the more advanced sample. Ca-No= Cancer tissue vs Normal tissue from FAP cases; Ad-No= Adenoma tissue vs Normal tissue from FAP cases; Ca-Ad= Cancer tissue vs Adenoma tissue from FAP cases; Ad-Ad= Adenoma tissue from FAP cases vs Adenoma tissue from FAP controls. For Ad-Ad comparison, number of samples in FAP case group and in FAP control group are shown. | | | | | | | | |

| **Supplemental Table 1: DEGs in Duodenal Neoplasia in FAP** | | | | | |
| --- | --- | --- | --- | --- | --- |
|  |  |  |  |  |  |
| **Aim** | **Comparison** | **# DEGs** | **# Protein Coding DEGs** | | **# Non-Protein Coding DEGs** |
|  |  |  | **Downregulated** | **Upregulted** |  |
| **Adenoma-Carcinoma Sequence in Cases** | Cancer-Normal | 169 | 57 | 64 | 48 |
|  | Adenoma-Normal | 25 | 14 | 5 | 6 |
|  | Cancer-Adenoma | 26 | 7 | 19 | 0 |
| **Cases vs Control Adenomas** | Adenoma-Adenoma | 18 | 13 | 0 | 5 |

| **Supplemental Table 2: List of DEGs in each comparison** | | | | | | | | |
| --- | --- | --- | --- | --- | --- | --- | --- | --- |
| **Cancer-Normal** | | | **Cancer-Adenoma** | | **Adenoma-Normal** | | **Adenoma-Adenoma** | |
| **Downregulated** | **Upregulated** | | **Downregulated** | **Upregulated** | **Downregulated** | **Upregulated** | **Downregulated** | **Upregulated** |
| ALDOB | SPTBN1 | BGN | DEFA5 | ANTXR1 | RBP2 | SNORD12B | ADH1C | SNORA76 |
| APOB | SNORD3D | LOC100507056 | DEFA6 | RAB31 | APOB | RNU105A | ADH4 |  |
| RBP2 | GAPDH | SNORD14E | ADH1C | VCAN | APOA4 | SNORA68 | ALDOB |  |
| APOA4 | HIST1H1E | MIR614 | CLCA1 | MMP2 | ALDOB | MMP1 | ANXA10 |  |
| SI | SOX9 | TXN | CPS1 | IL8 | TMPRSS15 | SNORA74A | APOA4 |  |
| TMPRSS15 | PGK1 | REG4 | MT1G | BGN | SLC26A3 | SNORD78 | C17orf78 | |
| SLC26A3 | KIF5B | SNORD41 | HMGCS2 | SPARC | GSTA1 | SNORD14A | CES1 |  |
| GIP | KPNA2 | SNORD70 |  | SCD | CYP3A4 | SLC12A2 | CLCA1 |  |
| MEP1B | SNORD3C | SNORA21 |  | COL6A3 | MEP1B | CEACAM6 | CPS1 |  |
| DEFA5 | MIR612 | SCD |  | GREM1 | SLC15A1 | REG4 | DMBT1 |  |
| GSTA1 | CD44 | GREM1 |  | CEACAM5 | MME | CEACAM5 | FABP1 |  |
| SLC15A1 | ANTXR1 | SNORD12C |  | COL1A1 | GSTA2 |  | LCT |  |
| CYP3A4 | SNORA65 | COL6A3 |  | COL1A2 | LCT |  | LOC399753 | |
| DEFA6 | IFITM1 | TMSB10 |  | COL12A1 | ASAH2C |  | MIR548O2 | |
| IGHM | SLC2A1 | SLC12A2 |  | COL3A1 |  |  | MIR548X | |
| GSTA2 | ANO1 | SNORD60 |  | SULF1 |  |  | RBP2 |  |
| SST | KIAA1199 | SNORD18A |  | POSTN |  |  | SI |  |
| ASAH2C | SNORD105B | SNORA74A |  | FN1 |  |  |  |  |
| MME | SNORD1A | SNORD14A |  | SPP1 |  |  |  |  |
| FOLH1 | VCAN | COL1A2 |  |  |  |  |  |  |
| ADH1C | RAB31 | COL3A1 |  |  |  |  |  |  |
| SLC5A1 | MIR622 | SNORD12B |  |  |  |  |  |  |
| FABP2 | ODC1 | COL1A1 |  |  |  |  |  |  |
| UGT2A3 | SNORA10 | SNORD16 |  |  |  |  |  |  |
| FOLH1B | SNORA76 | MMP1 |  |  |  |  |  |  |
| MTTP | SNORD97 | SULF1 |  |  |  |  |  |  |
| LCT | HOXB5 | COL12A1 |  |  |  |  |  |  |
| UGT2B17 | KRT18 | SNORD78 |  |  |  |  |  |  |
| MEP1A | RMRP | SNORD12 |  |  |  |  |  |  |
| CPS1 | SNORA71A | POSTN |  |  |  |  |  |  |
| PCK1 | TGFBI | FN1 |  |  |  |  |  |  |
| MS4A10 | HIST1H2BI | CEACAM6 |  |  |  |  |  |  |
| ACE2 | TNS4 | SNORD37 |  |  |  |  |  |  |
| AKR1B10 | IGHG4 | SPP1 |  |  |  |  |  |  |
| ADH4 | RNU12 | CEACAM5 |  |  |  |  |  |  |
| CYP2C19 | IFITM2 | SNORD83A |  |  |  |  |  |  |
| REG3A | THBS2 | IL8 |  |  |  |  |  |  |
| ASAH2 | YWHAZP3 | IFI27 |  |  |  |  |  |  |
| ADAMDEC1 | SNORA52 | MMP14 |  |  |  |  |  |  |
| CYP2C9 | MIR3687 | MMP12 |  |  |  |  |  |  |
| MGAM | PLP2 | SNORD92 |  |  |  |  |  |  |
| PDK4 | IFITM3 | RNU6ATAC |  |  |  |  |  |  |
| C19orf77 | TNC | SPARC |  |  |  |  |  |  |
| CREB3L3 | HIST1H2BK | MMP7 |  |  |  |  |  |  |
| GBA3 | SNORA68 | ANXA2P2 |  |  |  |  |  |  |
| ANPEP | MXRA5 | SNORA71C |  |  |  |  |  |  |
| DMBT1 | MMP2 | SNORD14B |  |  |  |  |  |  |
| ITLN2 | HSP90AB1 | |  |  |  |  |  |  |
| MT1H | GDF15 |  |  |  |  |  |  |  |
| LOC100653084 | RPL19 |  |  |  |  |  |  |  |
| SLC4A4 | PKM |  |  |  |  |  |  |  |
| LOC100134256 | DUOX2 |  |  |  |  |  |  |  |
| CYP2C18 | SNORD15A | |  |  |  |  |  |  |
| CLCA1 | THBS1 |  |  |  |  |  |  |  |
| SLC2A2 | SNORA31 | |  |  |  |  |  |  |
| LOC100507600 | SNORD126 | |  |  |  |  |  |  |
| HSD17B2 | TUBA1B | |  |  |  |  |  |  |
| ALDH1A1 | MYOF |  |  |  |  |  |  |  |
| SLC28A2 | RNU105A | |  |  |  |  |  |  |
| SLC6A19 | ANXA2 |  |  |  |  |  |  |  |
| DHRS11 | SNORD99 | |  |  |  |  |  |  |

| **Supplemental Table 3: FAP cases with undefined results on PCR** | | | | |
| --- | --- | --- | --- | --- |
|  |  |  |  |  |
| **DEG** | **Normal** | **Adenoma** | **Cancer** | **Adenoma(control)** |
| SPP1 | 5,9,10,11 | 10, 11 | 10 | None |
| CEACAM5 | 5,10,11 | 10, 11 | 10 | None |
| APOA4 | 10,11 | 10,11 | 10 | None |
| ANXA10 | 10,11,12 | 10,11 | 10 | None |
| SI | 10,11 | 6,9,10,11 | 3,4,6,8,9,10 | None |
| FAP case numbers for patients with undefined results on PCR verification, grouped by DEG. No FAP control had any undefined results | | | | |

| **Supplemental Table 4: Sample year and fixative for each FAP case** | | |
| --- | --- | --- |
|  |  |  |
| **FAP Case** | **Year** | **Fixative** |
| 1 | 2003 | FFPE |
| 2 | 2003 | FFPE |
| 3 | 2003 | FFPE |
| 4 | 2004 | FFPE |
| 5 | 2007 | FFPE |
| 6 | 2008 | FFPE |
| 7 | 2008 | FFPE |
| 8 | 2009 | FFPE |
| 9 | 2013 | FFPE |
| 10 | 2000 | Hollande's |
| 11 | 1999 | Hollande's |
| 12 | 1991 | Hollande's |
| FFPE= formalin-fixed paraffin-embedded sample | | |
